# Trajectory Analysis in FBG and the Incidence of Chronic Kidney Disease: A Nationwide Population-Based Study

**DOI:** 10.3390/biomedicines13020336

**Published:** 2025-02-01

**Authors:** Heewon Park, Ki Ryang Na, Yunkyeong Hwang, Suyeon Han, Kyungho Park, Hyerim Park, Eu Jin Lee, Young Rok Ham, Soon-Ki Ahn, Dae Eun Choi

**Affiliations:** 1 Department of Nephrology, Chungnam National University Hospital, Daejeon 35015, Republic of Korea; heewon910125@gmail.com (H.P.); drngr@cnu.ac.kr (K.R.N.); surejojo123@gmail.com (K.P.); eujinlee@cnuh.co.kr (E.J.L.); youngrok01@cnuh.co.kr (Y.R.H.); 2 Department of Nephrology, Daejeon Saint Mary’s Hospital, Catholic University of Korea, Daejeon 34943, Republic of Korea; hyk125@naver.com (Y.H.); sooyaa1208@gmail.com (S.H.); 3 Department of Medical Science, Medical School, Chungnam National University, Daejeon 35015, Republic of Korea; hye05240@gmail.com; 4 Department of Preventive Medicine, Chungnam National University Hospital, Daejeon 35015, Republic of Korea

**Keywords:** chronic kidney disease, fasting blood glucose, trajectory analysis

## Abstract

Objectives: This study aimed to classify fasting blood glucose (FBG) trajectories by sex and examine their associations with the risk of chronic kidney disease (CKD). Methods: Using data from the National Health Insurance Service-National Sample Cohort in Korea, participants aged 40 years and above, without CKD or diabetes mellitus (DM), were followed from 2002 to 2009. Based on their FBG trajectories, participants were categorized into two classes and stratified by sex. CKD incidence rates were analyzed according to these FBG trajectories, and the impact of additional risk factors on CKD incidence was assessed. Results: A total of 91,131 participants were analyzed. Among individuals classified in Class 1, FBG levels gradually increased from 90.7 (men) and 88.7 (women) in 2002 to 96.6 (men) and 93.2 (women) in 2009. In contrast, participants classified as Class 2 exhibited a rapid increase in FBG levels, rising from 106 (men) and 106 (women) in 2002 to 144 (men) and 132 (women) in 2009. The incidence of CKD increased over time in both men and women classified as Class 2 compared to Class 1, with respective hazard ratios (HR) of 1.35 for men and 1.53 for women. Additionally, increased age, hypertension, and body mass index (BMI) were independently associated with an elevated risk of CKD. Conclusions: The Class 2 group demonstrated a significantly higher incidence of CKD compared to the Class 1 group. This finding indicates the need for the proactive management of individuals with relatively high FBG levels featuring rapid FBG increases in order to mitigate the risk of CKD development.

## 1. Introduction

Chronic kidney disease (CKD) is increasingly recognized as a global health crisis, with over 850 million people affected worldwide as of 2022, and a prevalence rate surpassing 10% [1,2]. In Korea, the incidence of CKD is also on an upward trajectory [3]. CKD is a significant public health concern due to its potential progression to end-stage kidney disease (ESKD), as well as its association with higher rates of cardiovascular morbidity and mortality [4]. Early detection and intervention are essential for mitigating the economic and health impacts of CKD, making it critical in order to identify individuals at elevated risk.

Diabetes mellitus (DM) is a major contributor to ESKD, profoundly affecting patient quality of life and survival outcomes. Diabetic kidney disease (DKD), resulting from the structural and functional changes in the kidneys due to diabetes, is the leading cause of ESKD in developed nations, accounting for nearly 50% of cases [5]. The pathogenesis of DKD involves a complex interplay of genetic and environmental factors, leading to damage in the kidney’s filtration system [6]. Achieving optimal glycemic control is vital for preventing microvascular complications in DM patients; however, fluctuations in blood glucose levels present challenges in achieving accurate assessment.

The onset age of diabetes is gradually decreasing, and there is a growing prevalence of prediabetes, marked by impaired fasting glucose levels, particularly in early adulthood. In both the United States and Korea, the number of individuals with prediabetes is rising sharply, with Korea reporting a 25% prevalence among adults over 30 years of age [7,8,9]. Persistent elevation in blood glucose can indicate insulin resistance or impaired glucose tolerance, both of which are precursors to diabetes. Monitoring fasting blood glucose (FBG) levels regularly can help in the early detection of these abnormalities, allowing for timely lifestyle or medical interventions to prevent progression to diabetes and its complications, including DKD.

Although HbA1c is widely used to assess long-term glycemic control, its testing is often expensive and time-consuming, making it less practical for use in routine health checks, particularly for patients not diagnosed with DM [10]. Advances in continuous glucose monitoring (CGM) offer alternatives, but their high costs and limited indications restrict their widespread use [11,12]. Previous studies have explored the relationship between baseline blood glucose levels and CKD risk, yet individual glucose tests may not reliably reflect an individual’s glucose status due to variability in testing conditions [10,13]. Group-based trajectory modeling provides a method for identifying distinct patterns in FBG levels over time, offering valuable insights into metabolic health and CKD risk.

Given the established role of DM in CKD, our study focuses on the incidence of CKD in relation to glucose trends in individuals without a diabetes diagnosis. Prior research has highlighted sex-based differences in CKD prevalence and severity [14]. Population-based studies suggest that CKD epidemiology varies by sex, with a higher prevalence observed in women than in men, particularly in stage G3 CKD. Factors such as the natural decline in the Glomerular Filtration Rate (GFR) with age, potential over-diagnosis due to improper use of GFR equations, and the effect of longer life expectancy may contribute to higher CKD rates in women. Conversely, a greater number of men seem to start renal replacement therapy (RRT). This could be due to the effects of estrogen, which may offer some protection in women; the damaging impact of testosterone; or the adoption of less healthy lifestyles by men, which could lead to a more rapid decline in kidney function. Additionally, there is a tendency among older women to opt for conservative care rather than RRT [15,16].

This study aims to determine whether there are differential associations between glucose trends and CKD incidence when stratified by sex, using data from the Korean National Health Insurance Service database.

## 2. Materials and Methods

### 2.1. Data

The National Health Insurance Service (NHIS) of Korea, a non-profit organization responsible for providing health insurance to its citizens, covers approximately 97% of the population. The NHIS utilizes the Sample Cohort Database from the National Health Insurance Sharing Service (NHISS), which was established to facilitate the dissemination of national health data. This database includes a 2% sample of insured individuals and contains detailed information on medical resource utilization (e.g., consultations and medical check-ups), clinical statuses, and socio-economic variables, such as mortality and disability. The NHIS mandates regular health examinations for insured individuals based on their occupation, with exams conducted either annually or biennially. This study received approval from the Institutional Review Board of Chungnam National University Hospital (IRB number: 2024-02-015). The requirement for informed consent was waived due to the retrospective nature of the cohort study. We were granted access to the NHISS database on 12 May 2022 (NHIS-2022-02-340). In our analysis, we extracted estimated Glomerular Filtration Rate (eGFR) values from health checkup records. According to Korean health checkup regulations, eGFR should be calculated using the Modification of Diet in Renal Disease (MDRD) formula. Given the retrospective nature of the study, informed consent was not required from participants.

### 2.2. Study Population

**Figure 1:**
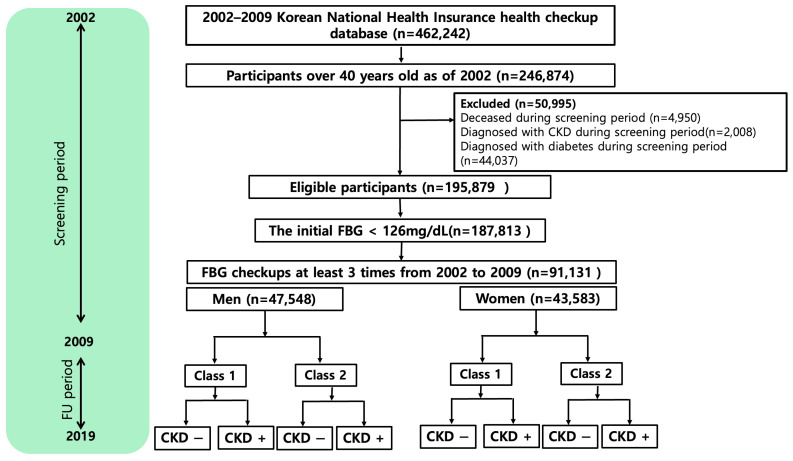
Flowchart of participant enrolment.

We included participants aged 40 years or older who underwent at least three health checkups between 2002 and 2009. Participants younger than 40 years were excluded due to the low incidence of CKD in this age group [15]. Given that diabetes is a well-established risk factor for CKD, we aimed to assess the risk of CKD among individuals without a diabetes diagnosis. To exclude potential diabetic patients, we only included individuals with initial FBG levels below 126 mg/dL. Additionally, to ensure the exclusion of individuals diagnosed with diabetes between 2002 and 2009, we excluded patients (*n* = 44,037) with insurance claims associated with diabetes diagnostic codes (E10, E11, E12, E13, E14). We also excluded individuals who died from any cause (n = 4950) during the screening period, as mortality terminated their observation. We excluded individuals diagnosed with CKD during the screening period (n = 2008). Among the 195,879 individuals screened, a cohort group was formed with those whose initial FBG during a screening period was less than 126 mg/dL (n = 187,813). Among these patients, it was confirmed that 51.5% underwent health checkups twice or less often over the 8-year period, 20.3% underwent checkups three times, 16.1% underwent checkups four times, 3.6% underwent checkups five times, 2.8% underwent checkups six times, 2.5% underwent checkups seven times, and 3.3% underwent checkups eight times. Of these, 91,131 participants who had received at least three health examinations from 2002 to 2009 were included in the analysis (Figure 1). The data were categorized and analyzed according to sex (men n = 47,585; women n = 43,583).

We identified new cases of CKD over a 10-year period from 2010 to 2019. CKD cases were identified using diagnostic codes, procedure codes, operation codes, and special case codes. The criteria for identifying CKD included the following:

- Patients with insurance claims under the diagnosis code N18X (International Classification of Disease, 10th Revision [ICD-10]; N181, N182, N183, N184, N185, N189).
- Patients who received prescriptions for peritoneal dialysis under procedure codes (O7071, O7072, O7073, O7074, O7076, O7077).
- Patients who underwent kidney transplantation (with insurance claims under the diagnosis code Z940 [kidney transplantation status] or prescriptions under the surgical code R3280 [renal transplantation]).
- Patients with insurance claims under the special case codes V001 (for maintaining hemodialysis for over 90 days), V003 (peritoneal dialysis), and V005 (kidney transplantation).

CKD incidence was analyzed based on new CKD diagnoses recorded in real time within the National Health Insurance Claim Data. Our study examined the incidence of new CKD diagnoses among individuals following health screenings. It is important to note that the diagnosis of CKD is not based on health checkup data but is confirmed by a nephrologist in a clinical setting, typically due to symptoms or other indications. The identification of comorbidities, such as hypertension, was based on insurance claims associated with hypertension diagnostic codes (I10, I11, I12, I13, I15).

Information regarding smoking history was collected through the medical checkup questionnaire. Data on income were classified into three levels—low (1st–3rd decile), middle (4th–7th decile), and high (8th–10th decile)—based on insurance premium deciles, provided by the NHISS Sampling Database. We included medical aid recipients in the low-income category.

### 2.3. Statistical Analysis

The statistical methodology employed in this study focuses on latent-class mixed modeling (LCMM) to analyze longitudinal FBG data. LCMM is utilized to identify latent subgroups within the population by detecting differences among patients. The time from disease onset, measured in months, serves as the key indicator for the analysis. The selection of the most appropriate model is guided by several criteria, including the Bayesian Information Criterion (BIC), a high mean posterior probability score (ideally above 0.7), sufficient class sizes, and the clinical relevance of the models. To ensure adequate sample sizes and maintain clinical relevance, this study explores models with group sizes ranging from two to four classes.

Baseline comparisons among these subgroups are conducted by using the Student’s *t*-test for continuous variables and the chi-square test for categorical variables. The cumulative incidence of new-onset CKD was calculated by the product limit (Kaplan–Meier) method of survival probability and compared across the groups using the Kaplan–Meier method and the log-rank test. Multivariate analysis was performed via Cox proportional hazards analysis to assess the associations of the latent class with the development of CKD. The proportional hazards assumption was evaluated graphically by log–log plots, and there was no significant departure over time. The hazard ratio (HR) and 95% confidence interval (CI) were estimated. The final model included the interaction term (age group and BMI group) for men and the interaction term (age group and BMI group, age group and HTN in 2009, and BMI group and HTN in 2009) for women, and the significant interactions were detected. Variable selection was performed by stepwise methods, which included both forward selection and backward elimination.

Statistical analysis was conducted using R software (version 4.3.0; R Foundation for Statistical Computing, Vienna, Austria), with the “LCMM” package specifically employed for latent-class analysis. The adjusted hazard ratios were estimated and plotted with the “survival” and the “survminer” packages. A *p*-value of less than 0.05 was considered statistically significant.

### 2.4. FBG Trajectory

**Figure 2:**
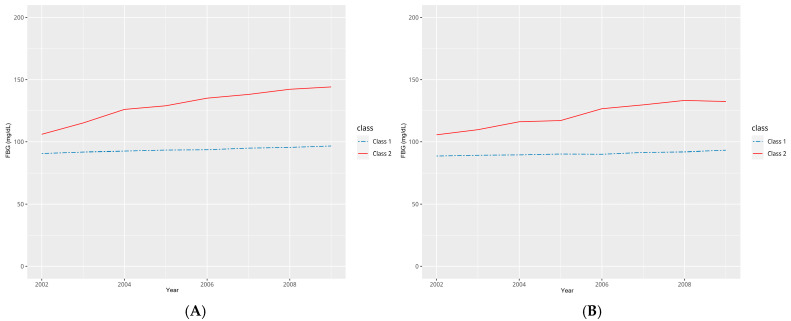
Change in FBG levels during screening period by trajectory groups by sex: (**A**) men; (**B**) women.

We analyzed the FBG trajectory patterns from 2002 to 2009, with data stratified by sex (Figure S1). Considering the BIC and sample size for the number of classes, we determined that dividing the data into two classes was the most appropriate approach for further analysis. In detail, FBG trajectory analysis using LCMM was carried out through a three-step process. In the first step, it was assumed that the FBG trajectories could be classified into 2–4 classes based on prior knowledge. In the second step, each trajectory was fitted to different shapes, including linear and quadratic forms. Then, the model fit was compared using BIC across various numbers of trajectories. Following this, the most appropriate model was selected by combining knowledge and statistical considerations. In the third step, model selection was performed. The criteria used for model selection were as follows: (1) A lower BIC value indicated a better model fit. (2) The average posterior probability of class membership for everyone assigned to a class was required to exceed 0.7. (3) The sample size for each class was required to be greater than 2.5%. Finally, based on the above criteria, we determined that the most suitable model was the linear trajectory model with two classes (Table S1). Class 1 shows a relatively stable and lower FBG level over time, remaining close to 100 mg/dL. Class 2 starts at a higher baseline FBG level (around 110 mg/dL) and shows a gradual increase over the years (Figure 2). This indicates that Class 2 had FBG levels of 100 mg/dL or higher from the beginning of the screening period, suggesting the impairment of fasting glucose and a prediabetic state.

## 3. Results

In this study, a total of 91,131 participants were included in the analysis, comprising 47,548 men (52%) and 43,583 women (48%). Participants were categorized into groups based on the trajectory of their FBG, and their characteristics are summarized in Table 1. The average age across the classes ranged from 50.8 to 54.6 years, with the highest proportion of participants being between 45 and 54 years old in all classes. When examining the proportions related to income levels, the high-income group had the highest proportion across almost all classes. However, Class 1 had a larger proportion of individuals in the high-income category compared to Class 2. A significant sex disparity was observed in smoking history: among women, the proportion of nonsmokers was overwhelmingly high in both classes (Class 1 97.2%, Class 2 97.0%). In contrast, among men, the proportions for each smoking status were similar across the two classes. In Class 2, regardless of sex, a higher proportion of participants had a BMI of 25 or above compared to the other BMI groups (men 45.1%, women 47.8%). Regarding baseline hypertension, Class 1 had a higher proportion of participants without hypertension (men 58.0%, women 58.5%), whereas Class 2 had a higher proportion of participants with a history of hypertension, regardless of sex (men 58.8%, women 66.3%). When comparing the average blood pressure of each class in 2002 and 2009, Class 2 showed higher systolic blood pressure (SBP) and diastolic blood pressure (DBP) than Class 1.When comparing eGFR by class in 2009, it was found that men had higher eGFR levels than women. In 2009, the eGFR for men was 78.6 mL/min/1.73 m^2^ in Class 1, and it was 81.9 mL/min/1.73 m^2^ in Class 2. For women, the eGFR in 2009 was 72.3 mL/min/1.73 m^2^ in Class 1, and it was 73.3 mL/min/1.73 m^2^ in Class 2. We also examined the annual average FBG levels during the screening period. In both sexes, the average values for Class 2 increased over time. Although there were years when the average exceeded 126 mg/dL, this value represented the average during the screening period, and individuals with an initial FBG exceeding 126 mg/dL were not enrolled in the cohort for that year. During the follow-up period, the incidence of CKD was 3.56% in Class 1 men and 6.39% in Class 2 men. For women, the incidence was 2.27% in Class 1 and 4.34% in Class 2. In men, the average follow-up period was 114 months for Class 1 and 111 months for Class 2. In women, the average follow-up period was 117 months for Class 1 and 114 months for Class 2.

**Figure 3:**
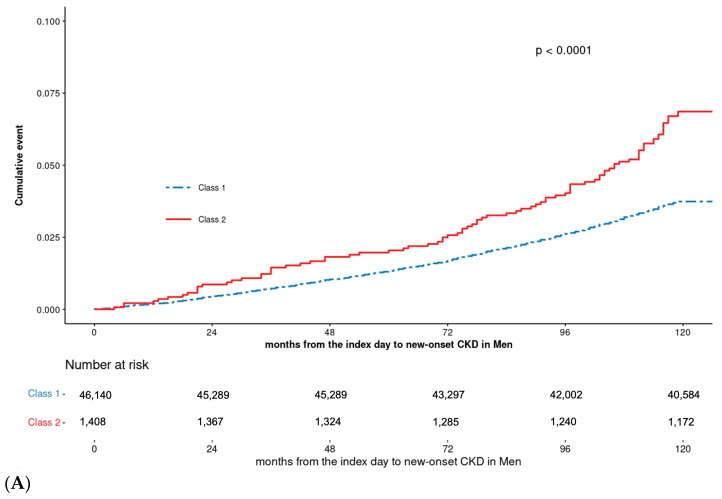

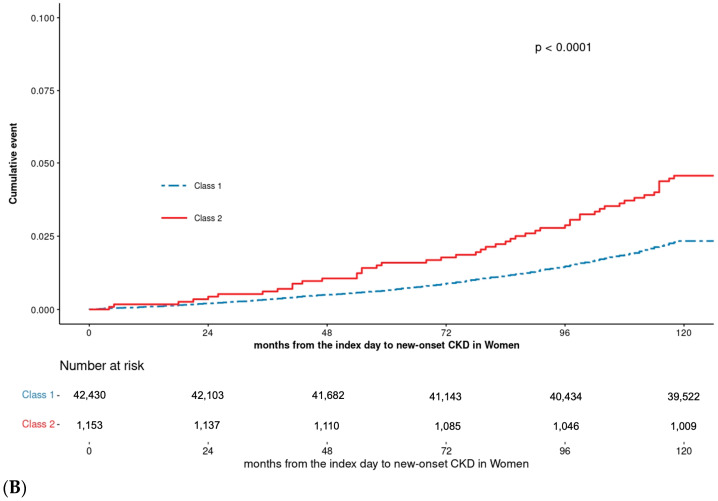
Kaplan–Meier curves for incidence of CKD among 2 classes: (**A**) men and (**B**) women.

**Figure 4:**
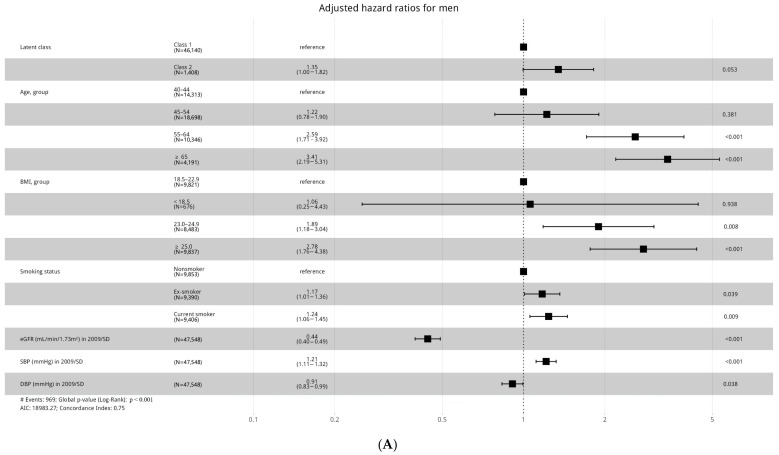

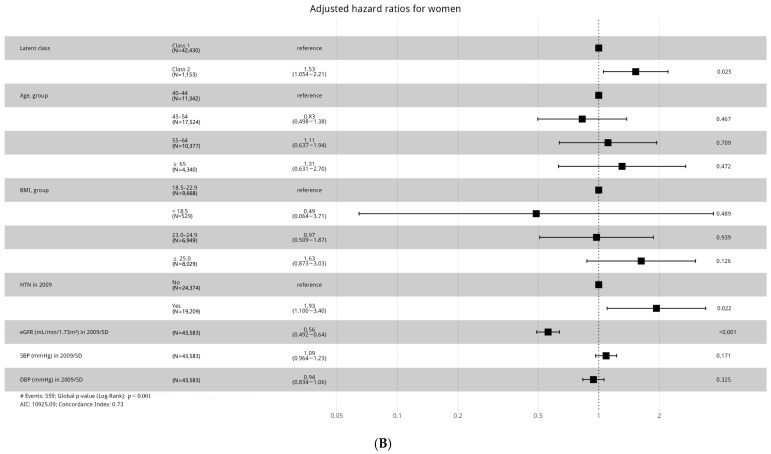
Hazard ratios (95% confidential interval) of incidence of CKD according to various clinical factors in men (**A**) and women (**B**). (**A**) was adjusted for age, BMI, smoking, income, eGFR, blood pressure, and the interaction term (age group and BMI group). (**B**) was adjusted for age, BMI, smoking, income, eGFR, blood pressure, HTN in 2009, and the interaction terms (age group and BMI group, age group and HTN in 2009, and BMI group and HTN in 2009). Abbreviations: BMI; body mass index.

CKD incidence rates over time were analyzed for each sex within the classes, with both sexes showing an increased CKD incidence in Class 2 over time (Figure 3). Adjusted hazard ratios, accounting for potential confounding factors influencing CKD, are presented in Figure 4, revealing sex-specific differences. In men, the risk of incidence of CKD was higher in Class 2 compared to Class 1, with a hazard ratio of 1.35. In women, the hazard ratio for CKD incidence was 1.53 in Class 2 compared to Class 1, indicating a higher risk than in men. The risk of CKD also increased for men with advancing age. Compared to the group of men under 44 years old, the hazard ratio for CKD incidence was 3.41 times higher in men aged 65 and older. In contrast, women showed different results compared to men. Among women, the hazard ratio for CKD incidence in the group aged 65 years and older was 1.31 compared to the youngest group. However, based on the *p*-value and the hazard ratios for other groups, no statistically significant results were observed. In men, the risk of CKD incidence was 1.06 times higher in the underweight group (BMI < 18.5), 1.89 times higher in the overweight group (BMI 23–24.9), and 2.78 times higher in the obese group (BMI ≥ 25) compared to the normal weight group (BMI 18.5–22.9). Among women, compared to the normal weight group, the hazard ratio for CKD incidence was 0.49 in the underweight group, 0.97 in the overweight group, and 1.63 in the obese group; however, none of these results were statistically significant. The relationship between BMI and CKD incidence in men follows a U-shaped pattern, with the hazard ratio increasing as BMI deviates from the normal range. Smoking history analysis revealed that in men, the risk of CKD increased progressively from nonsmokers to ex-smokers (HR = 1.17) and current smokers (HR = 1.24). Among women, when examining the hazard ratio for CKD incidence based on the presence of hypertension in 2009, the group with hypertension had a higher risk of CKD incidence, with a hazard ratio of 1.93 compared to the group without hypertension.

We standardized the indicators by dividing them by their standard deviations (SD) to enhance the reliability of the aHR values. Regarding the relationship between eGFR and CKD incidence, a 1 SD increase in eGFR was associated with a 56% reduction in CKD incidence among men and a 44% reduction among women. In contrast, for SBP, a 1 SD increase was associated with a 21% increase in CKD risk in men. Among men, from the perspective of DBP, each 1 SD increase was associated with a 9% reduction in the risk of CKD incidence. In contrast, among women, neither SBP nor DBP showed a significant association with CKD incidence.

## 4. Discussion

This study aimed to assess the relationship between FBG trajectories and the incidence of CKD across a large cohort of 91,131 participants. By analyzing participant groups based on their FBG trajectories and their subsequent CKD risk over time, we identified several important findings with significant implications for public health strategies and clinical practice, particularly regarding gender differences, BMI, smoking history, and hypertension.

In this study, we expanded our analysis beyond a single FBG measurement to assess CKD onset. Instead, we adopted a longitudinal approach to examine FBG trajectories over an extended period, categorizing them into distinct classes. This study is notable for its use of a representative cohort of relatively young individuals and for estimating CKD incidence risk over time through latent-class analysis of FBG changes. Beyond the well-established link between diabetes and CKD, numerous studies have explored the relationship between undiagnosed diabetes, prediabetes, and CKD. For instance, the KORA F4-Study (2018) demonstrated an association between low eGFR and various prediabetic stages [15]. In our study, individuals whose FBG levels were ≥100 mg/dL in 2002 and maintained impaired fasting glucose levels (Class 2) during the screening period exhibited a higher risk of developing CKD during the follow-up period compared to those with stable FBG levels (Class 1, Figure 3). This finding aligns with prior studies linking hyperglycemia and glycemic variability to renal function decline. These factors are possibly mediated by mechanisms such as oxidative stress, inflammation, and endothelial dysfunction [17,18,19]. These results underscore the importance of early detection and the management of rising FBG levels to prevent CKD onset.

The findings demonstrate a clear sex disparity in the risk of CKD incidence, with women showing a higher adjusted hazard ratio (aHR) for CKD in Class 2 compared to men. While the hazard ratio for men in Class 2 was 1.35, the hazard ratio for women in Class 2 was 1.53, suggesting a greater risk of CKD in women, despite both classes showing an increasing CKD incidence over time. This aligns with prior studies suggesting that women may be more susceptible to kidney damage or experience faster CKD progression under specific risk factors, such as FBG abnormalities [16]. The observation that women had a higher hazard ratio for CKD incidence than men aligned with earlier research suggesting the importance of sex differences in CKD pathophysiology [20,21]. Possible explanations include hormonal factors, differences in kidney structure and function, or disparities in healthcare utilization and disease awareness [20,22]. However, our study also noted that women had lower baseline eGFR levels than men, which may partly explain their higher susceptibility.

Interestingly, the risk of CKD in men increased significantly with age, with aHR for CKD incidence being 3.41 times higher in men aged 65 years and older compared to those under 44 years old. In contrast, among women, the risk did not increase as dramatically with age, and the difference in hazard ratios between the age groups was not statistically significant. This points to the potentially more age-dependent effect of CKD risk in men than in women, which warrants further exploration, especially in aging populations where CKD burden is expected to increase [23].

The relationship between BMI and CKD incidence varied notably between men and women. In men, a U-shaped association was observed, with the risk of CKD increasing progressively from underweight (BMI < 18.5) to overweight (BMI 23–24.9) and obese (BMI ≥ 25) categories. This pattern highlights the heightened risk of CKD among men who are either underweight or overweight/obese, indicating that both extremes of BMI may contribute to kidney damage. Both underweight and obese individuals exhibited an increased risk of CKD, potentially due to factors like malnutrition, metabolic syndrome, and increased systemic inflammation [24]. Additionally, adiposity, which is associated with an adverse cardiovascular risk profile, was also linked to an increased risk of CKD in our study, corroborating the results of previous research [25]. Obesity can directly injure glomeruli through hemodynamic changes, primarily due to afferent arteriole vasodilation and increased proximal tubular salt reabsorption. These changes result in glomerular hyperfiltration, which eventually leads to proteinuria [25]. The finding reinforces the importance of maintaining a healthy weight to prevent CKD, as both being both underweight and of an excessive weight are risk factors for kidney disease in men. In women, however, the relationship between BMI and CKD was less clear. While the hazard ratio for CKD incidence was higher in the obese group (1.63 compared to normal weight), it was not statistically significant. The lack of a strong association between BMI and CKD in women may reflect differing metabolic pathways, hormonal influences, or potentially unmeasured confounders that affect women’s risk of CKD differently from men. These gender-specific differences in BMI-CBD risk should be investigated in future research.

Hypertension emerged as one of the most robust risk factors for CKD, with individuals having a history of hypertension in 2009 exhibiting significantly higher risk of developing CKD in both men and women. For women, the hazard ratio for CKD incidence was 1.93 in the hypertensive group compared to the normotensive group, emphasizing the need for the early intervention and management of hypertension in women to reduce the long-term risk of kidney disease. This is consistent with the existing literature that underscores the role of hypertension in kidney dysfunction, particularly in populations with abnormal glucose metabolism [26].

The analysis of FBG trajectories revealed a concerning trend in both sexes, with the average FBG increasing over time in Class 2. Even though participants with an initial FBG greater than 126 mg/dL were excluded from the cohort, indicating that those with overt diabetes were not included, the continuous increase in FBG levels was associated with higher CKD incidence. The fact that the average FBG exceeded 126 mg/dL during the screening period, particularly in Class 2, highlights the critical importance of managing blood glucose levels within the normal range to reduce the risk of CKD.

The study further explored the relationship between eGFR and CKD incidence, which revealed significant findings. A 1 SD increase in eGFR was associated with a 56% reduction in CKD incidence in men and a 44% reduction in women. This suggests that maintaining better kidney function, as reflected by higher eGFR levels, can significantly reduce the risk of developing CKD. In contrast, a higher SBP was linked to increased CKD risk in men, with each 1 SD increase in SBP associated with a 21% increase in CKD risk, while DBP was inversely related to CKD risk in men. These findings suggest that in addition to controlling FBG and hypertension, preserving kidney function through monitoring eGFR and managing blood pressure is crucial for preventing CKD.

Smoking history also emerged as an important factor in CKD risk, particularly in men. The study found a progressive increase in CKD risk from nonsmokers to ex-smokers and current smokers. This aligns with the known adverse effects of smoking on kidney function, which likely contributes to the observed increased risk of CKD in smokers. While the results for women were not as strong, this highlights the potential of smoking cessation interventions as part of a comprehensive CKD prevention strategy.

In this context, our study is significant as it utilizes the NHISS Cohort data, representing the Korean population, to investigate relatively young individuals. One of the strengths of this study is its use of the NHISS Cohort database, a large-scale dataset that enables longitudinal cohort studies of the entire Korean population over an extended period. Korea’s National Health Insurance system is globally recognized. Because it encompasses the entire population, the sample data from the National Health Insurance Service—constructed through stratified systematic sampling based on medical records, examination results, residency, insurance premiums, and healthcare institution information—can be considered representative of health information for Koreans. Furthermore, this study is distinguished by its creation of a new cohort spanning eight years, starting from individuals who underwent health screenings in 2002 and were sampled from the eligibility database, despite the data’s inherent individualistic or fragmented structure.

Another strength of the study lies in its novel use of group-based trajectory modeling, which considers both the trends in variables over time and the size of the study population. This statistical method appropriately groups individuals based on their trajectories and allows for the estimation of CKD incidence risk over eight years according to changes in FBG levels. If the group-based trajectory modeling method is further utilized to identify distinctive patterns of change during the long-term monitoring of fasting blood glucose levels, it could aid in establishing preventive measures before disease onset. Additionally, it could help in understanding the differences in the actual incidence patterns of CKD. This approach may provide valuable insights into disease prediction. Previous applications of group-based trajectory modeling in epidemiological research primarily targeted specific age groups or single time points, grouping trajectories over fixed periods and analyzing their relationship to disease-related indicators. In contrast, this study is distinctive in estimating the risk of CKD occurrence according to groupings based on FBG trajectories over the entire cohort period of individuals aged 40 and above.

We identified the limitations of this study. We were unable to account for individuals taking medications that could elevate blood glucose levels (e.g., glucocorticoids, beta agonists, megestrol) because medication history was not available in the database. Among individuals who underwent health checkups, those who participated in at least three out of eight checkups were included in the analysis. As a result, the dataset does not represent complete balance data for all eight checkups. This limitation may have constrained the analysis, particularly in partially trajectory-based evaluations, as it does not fully capture the overall variability within the population. Additionally, as the study was conducted solely on the South Korean population, the data lack diversity in terms of multi-ethnic representation. This limitation poses challenges in generalizing the findings to broader, more diverse populations.

## 5. Conclusions

Our findings emphasize the need for early interventions targeting glycemic control, weight management, and blood pressure optimization, particularly in individuals with high or increasing FBG levels. Sex-specific strategies may also be necessary, given the differing risk profiles observed between men and women. Additionally, the study underscores the potential utility of monitoring FBG trajectories as a predictive marker for CKD risk.

## Figures and Tables

**Table 1:** Baseline characteristics of study participants by sex.

		Men			Women	
	Class 1 N = 46,140	Class 2 N = 1408	*p*.overall	Class 1 N = 42,430	Class 2 N = 1153	*p*.overall
Age, 2002	50.8 (8.72)	51.2 (8.31)	0.094	51.5 (8.73)	54.6 (9.36)	<0.001
Age, group in 2002:			0.004			<0.001
40–44	13,944 (30.2%)	369 (26.2%)		11,165 (26.3%)	177 (15.4%)	
45–54	18,093 (39.2%)	605 (43.0%)		17,079 (40.3%)	445 (38.6%)	
55–64	10,028 (21.7%)	318 (22.6%)		10,046 (23.7%)	331 (28.7%)	
≥65	4075 (8.83%)	116 (8.24%)		4140 (9.76%)	200 (17.3%)	
Income status in 2009:			0.012			0.054
Low	9063 (20.0%)	296 (21.3%)		10,520 (25.1%)	280 (24.6%)	
Middle	15,506 (34.2%)	514 (36.9%)		14,447 (34.4%)	430 (37.8%)	
High	20,779 (45.8%)	582 (41.8%)		16,989 (40.5%)	429 (37.7%)	
Smoking status in 2009:			0.086			0.885
Nonsmoker	9553 (34.5%)	300 (31.8%)		23,583 (97.2%)	689 (97.0%)	
Ex-smoker	9087 (32.8%)	303 (32.2%)		264 (1.09%)	7 (0.99%)	
Current smoker	9067 (32.7%)	339 (36.0%)		427 (1.76%)	14 (1.97%)	
BMI, group in 2009:			<0.001			<0.001
<18.5	663 (2.38%)	13 (1.37%)		515 (2.11%)	14 (1.96%)	
18.5–22.9	9581 (34.4%)	13 (1.37%)		9475 (38.7%)	193 (27.1%)	
23.0–24.9	8216 (29.5%)	267 (28.2%)		6784 (27.7%)	165 (23.1%)	
≥25.0	9409 (33.8%)	428 (45.1%)		7688 (31.4%)	341 (47.8%)	
HTN in 2009:			<0.001			<0.001
No	26,766 (58.0%)	580 (41.2%)		23,985 (56.5%)	389 (33.7%)	
Yes	19,374 (42.0%)	828 (58.8%)		18,445 (43.5%)	764 (66.3%)	
SBP (mmHg) in 2002	127 (16.0)	132 (17.3)	<0.001	122 (17.5)	131 (20.9)	<0.001
SBP (mmHg) in 2009	126 (14.6)	130 (15.7)	<0.001	123 (15.5)	129 (16.7)	<0.001
DBP (mmHg) in 2002	80.7 (10.8)	84.3 (11.1)	<0.001	76.3 (11.5)	80.9 (12.1)	<0.001
DBP (mmHg) in 2009	78.8 (9.72)	80.7 (10.2)	<0.001	76.0 (9.92)	78.5 (9.84)	<0.001
eGFR (mL/min/1.73 m^2^) in 2009	78.6 (21.0)	81.9 (33.1)	0.003	72.3 (20.8)	73.3 (24.6)	0.287
FBG (mg/dL), 2002	90.7 (12.5)	106 (13.4)	<0.001	88.7 (11.7)	106 (12.3)	<0.001
FBG (mg/dL), 2003	91.9 (13.5)	115 (24.3)	<0.001	89.3 (11.8)	110 (17.4)	<0.001
FBG (mg/dL), 2004	92.5 (13.8)	126 (31.0)	<0.001	89.6 (12.0)	116 (22.9)	<0.001
FBG (mg/dL), 2005	93.3 (13.9)	129 (30.7)	<0.001	90.3 (11.9)	117 (22.0)	<0.001
FBG (mg/dL), 2006	93.6 (13.4)	135 (31.3)	<0.001	90.1 (11.8)	127 (26.9)	<0.001
FBG (mg/dL), 2007	94.9 (13.8)	138 (33.4)	<0.001	91.6 (11.6)	130 (28.3)	<0.001
FBG (mg/dL), 2008	95.5 (13.8)	142 (34.0)	<0.001	91.9 (11.6)	133 (29.6)	<0.001
FBG (mg/dL), 2009	96.6 (14.1)	144 (34.0)	<0.001	93.2 (11.7)	132 (29.9)	<0.001
Incident of CKD:			<0.001			<0.001
No	44,497 (96.4%)	1318 (93.6%)		41,465 (97.7%)	1103 (95.7%)	
Yes	1318 (93.6%)	90 (6.39%)		1103 (95.7%)	50 (4.34%)	
Follow up month for CKD	114 (21.3)	111 (25.1)	<0.001	117 (15.0)	114 (20.8)	<0.001

*p* < 0.001, indicating that statistical significance is achieved due to the large sample size.

## Data Availability

The original contributions presented in the study are included in the article/Supplementary Material, further inquiries can be directed to the corresponding authors.

## Supplementary Materials

The following supporting information can be downloaded at: https://www.mdpi.com/article/10.3390/biomedicines13020336/s1, Figure S1. Spaghetti plot of fast blood glucose plotted from 2002 to 2009. (A) men (B) women. Table S1. Model fit evaluation information for each LCMM tested.
