# Supplementary material for: Trajectory Analysis in FBG and the Incidence of Chronic Kidney Disease: A Nationwide Population-Based Study
Source: Biomedicines. 2025 Feb 1;13(2):336. doi: 10.3390/biomedicines13020336 (PMC11852470; doi:10.3390/biomedicines13020336)
Supplement: Supplementary file 1 [file biomedicines-13-00336-s001.zip › biomedicines-3446779-supplementary.pdf]

A

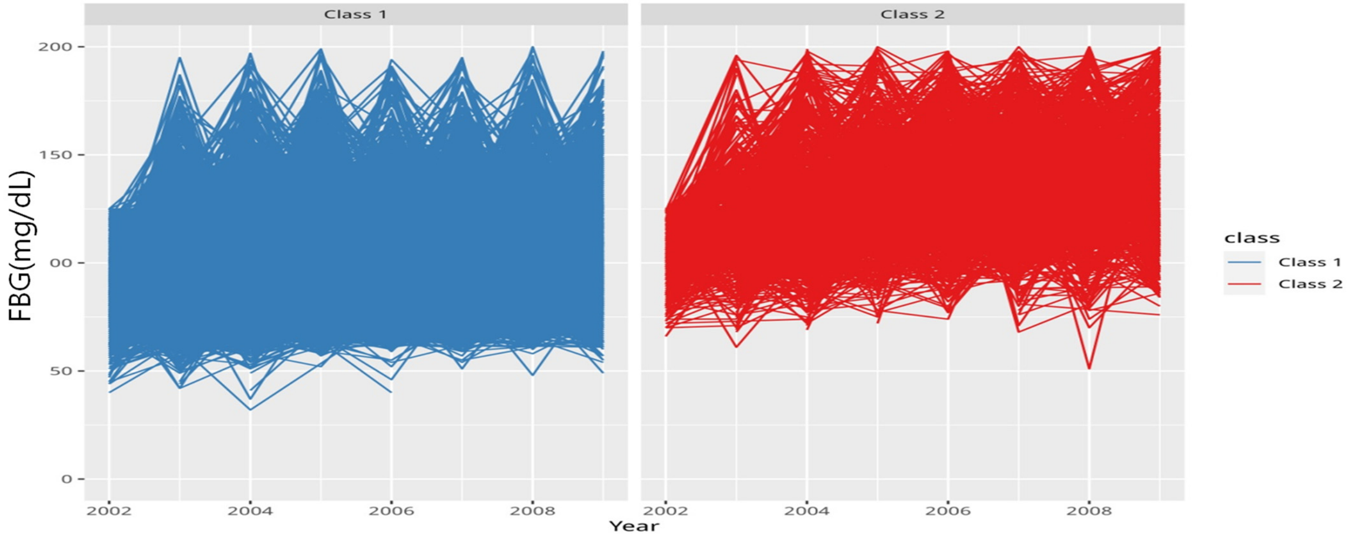

B

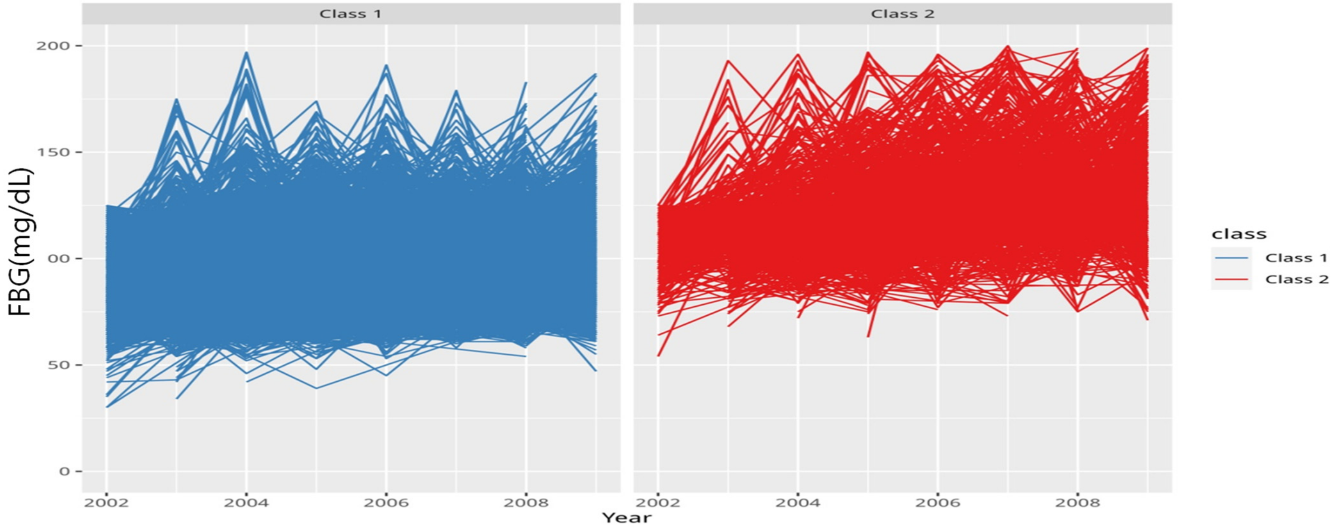

Figure S1. Spaghetti plot of fast blood glucose plotted from 2002 to 2009. (A) men (B) women

Table S1. Model fit evaluation information for each LCMM tested

Latent class number of the model	Polynomial degree	Men					Women				
		Maximum log-likelihood	BIC	% participants per class	Mean posterior probabilities	% Posterior probabilities > 70%	Maximum log-likelihood	BIC	% participants per class	Mean posterior probabilities	% Posterior probabilities > 70%
**Two-class**	**Linear**	**-887728.9**	**1775512**	**2.96/97.04**	**0.89/0.99**	**84.59/99.45**	**-653466.2**	**1306986**	**2.65/97.35**	**0.88/0.99**	**81.96/99.25**
	Quadratic	-864598.6	1729305	27.27/72.73	0.52/0.53	0/0	-640512.3	1281131	4.81/95.19	0.58/0.74	5.67/67.44
Three-class	Linear	-887728.9	1775533	3.04/96.96/0	0.88/0.85/NA	82.48/98.87/NA	-653466.2	1307007	2.74/97.26/0	0.87/0.95/NA	79.1/99/19/NA
	Quadratic	-864236.8	1728603	10.22/89.78/0	0.64/0.59/NA	34.60/NA/NA	-640262.9	1280654	4.6/95.4/0	0.67/0.75/NA	41.19/86.17/NA
Four-class	Linear	-886881.0	1773859	6.28/92.8/0.91/0	0.78/0.92/0.91/NA	66.77/96.97/88.94/NA	-652682.1	1305460	1.48/89.07/0.16/9.29	0.85/0.93/0.89/0.72	78.26/93.83/88.73/56/27
	Quadratic	-864388.9	1728929	29.58/70.42/0/0	0.47/0.31/NA/NA	13.42/0/NA/NA	-640262.9	1280675	6.54/0/93.46/0	0.58/Na/0.48/NA	28.53/NA/0/NA

BIC, Bayesian information criteria; LCMM, latent class mixed model.
The best fitting model is highlighted in bold characters.
